# International Veterinary Epilepsy Task Force consensus reports on epilepsy definition, classification and terminology, affected dog breeds, diagnosis, treatment, outcome measures of therapeutic trials, neuroimaging and neuropathology in companion animals

**DOI:** 10.1186/s12917-015-0460-3

**Published:** 2015-08-28

**Authors:** Holger A. Volk

**Affiliations:** Department of Clinical Science and Services, Royal Veterinary College, Hatfield, AL9 7TA Hertfordshire, UK

**Keywords:** Epilepsy, Seizures, Dog, Classification, Semiology

## Editorial

Epilepsy is one of the most common chronic neurological diseases in companion animals. Its prevalence has been estimated to be 0.6-0.75 % in the general dog population [1, 2], which means that nearly every 130^th^ dog presenting in a veterinary practice will have epilepsy. Dogs and cats with epilepsy experience debilitating epileptic seizures, but epilepsy causes more than recurrent epileptic seizures alone. Patients with epilepsy can suffer from transient postictal behavioural changes and/or clinical deficits. Furthermore, affected dogs have a shortened life expectancy, and are at an increased risk of developing comorbidities affecting the interictal period, such as neurobehavioural changes, and a reduced quality of life [3–6]. The impact of the disease does not only affect the patient, but also affects the quality of life of the pet’s owner [5–7]. Based on the importance of epilepsy for clinical and especially neurological practice a flourish of studies have been performed and published over the last 30 years.

Despite this plethora of new information, the classifications, definitions, terminology, therapeutic outcome measures, neuroimaging and neuropathological standards have differed between many of these studies, making it difficult to draw comparisons. This could potentially limit their scientific impact. Furthermore, this has prevented the implementation of a common understanding of epilepsy and standardized professional guidelines, which could support clinicians when diagnosing companion animals with epilepsy and advising their owners. Many of the classifications, definitions and terminology used have reflected the most recent proposals of that time of an international human epilepsy organisation, the International League Against Epilepsy (ILAE). Since the 1960s the ILAE have worked on defining, classifying and agreeing on the terminology used in human epilepsy [8–14]. The ILAE sees itself as “*the world*'*s preeminent association of physicians and other health professionals working towards a world where no persons*' *life is limited by epilepsy*” and has the mission “*to ensure that health professionals*, *patients and their care providers*, *governments*, *and the public world*-*wide have the educational and research resources that are essential in understanding*, *diagnosing and treating persons with epilepsy*” (ILAE homepage www.ilae.org). The ILAE builds taskforces to fulfil its mission, resulting in the publication of consensus statements to provide the scientific and clinical framework for the epilepsy community. These consensus statements are regularly reviewed every 5-10 years, reflecting the constant improvements in our understanding of the disease, its treatment, comorbidities and complications.

In 2014, a group of Veterinary Neurology Specialists and Non-specialists founded the International Veterinary Epilepsy Task Force (IVETF). The IVETF is deliberately independent to any other veterinary or human organisation and its main aim is to provide the veterinary community, breeders and dog (and in part cat) owners with consensus statements on the key areas in the field of epilepsy. There is a ‘chain of care’ from the animal’s breeder and owner through the first opinion practitioner to the neurology specialist and neuroscientist. Each consensus statement aims to be a ‘user friendly’, pragmatic, reliable and valid tool for the benefit of all of these stakeholder groups. Furthermore, IVETF has worked towards building a scientific and clinical framework to manage and research epilepsy appropriately, and to provide a platform to enable communication with other stakeholders using the same, agreed “common language”. Each consensus statement is based upon the current published understanding of epilepsy and represents in parts the authors’ interpretation of the published evidence. IVETF reflected new thoughts from the human ILAE, but also considered well accepted veterinary terminology and practice. To ensure we had a broad range of stakeholders involved, the consensus-working group was composed of and/or consulted veterinary and human neurologists and neuroscientists, practitioners, neuropharmacologists and neuropathologists. It is the first time that so many clinicians and scientists have united and formally agreed on the key aspects of companion animal epilepsy. This culminated in twenty-six co-authors being involved in the process of developing seven consensus statements. These consensus statements should be seen as the beginning rather than the end of this process, with the IVETF planning to broaden its remit and membership in the future.

The IVETF has agreed on the following consensus statements. The IVETF hopes that each statement will help advance the field of canine and feline epilepsy and ultimately lead to better care for our patients:

1. International Veterinary Epilepsy Task Force consensus report on epilepsy definition, classification and terminology in companion animals
2. International Veterinary Epilepsy Task Force Consensus Proposal: Diagnostic approach to epilepsy in dogs
3. International Veterinary Epilepsy Task Force current understanding of idiopathic epilepsy of genetic or suspected genetic origin in purebred dogs
4. International Veterinary Epilepsy Task Force consensus proposal: Medical treatment of canine epilepsy in Europe
5. International Veterinary Epilepsy Task Force Consensus Proposal: Outcome of therapeutic interventions in canine and feline epilepsy
6. International Veterinary Epilepsy Task Force recommendations for a veterinary epilepsy-specific MRI protocol
7. International Veterinary Epilepsy Task Force recommendations for systematic sampling and processing of brains from epileptic dogs and cats

## Abbreviations

IVETF: International Veterinary Epilepsy Task Force
ILAE: International League Against Epilepsy
